# Herding in human groups is related to high autistic traits

**DOI:** 10.1038/s41598-020-74951-8

**Published:** 2020-10-21

**Authors:** I. Z. Marton-Alper, H. Z. Gvirts-Provolovski, M. Nevat, M. Karklinsky, S. G. Shamay-Tsoory

**Affiliations:** 1 Department of Psychology, University of Haifa, Haifa, Israel; 2 Department of Behavioral Sciences and Psychology, Ariel University, Ariel, Israel; 3 Department of Computer Science and Applied Mathematics, Weizmann Institute of Science, Rehovot, Israel; 4 Integrated Brain and Behavior Research Center (IBBRC), Haifa, Israel

**Keywords:** Human behaviour, Social behaviour, Psychology

## Abstract

Herding is ubiquitous throughout all social life forms, providing beneficial outcomes. Here, we examine whether herding emerges spontaneously in human groups and whether it adheres to the core principles of herding observed in the animal kingdom. Using a computerized paradigm involving the movements of circles, we tested the emergence of spontaneous and intentional herding of 136 participants assigned into groups of four participants. Herding was assessed by measuring directional synchrony in the movements of the circles, level of cohesion, and separation between circles. We found that human groups tend to spontaneously herd, particularly in terms of directional synchrony, supporting the notion of a human herding instinct. We further asked whether individuals with high traits of Autism Spectrum Disorder (ASD) exhibit differences in their herding tendencies. Results indicated that individuals with high ASD traits showed greater social separation from the group, compared to individuals with low ASD traits. Moreover, we found diminished spontaneous synchrony, but intact instructed synchrony in the high vs. the low ASD traits group. We contend that humans spontaneously herd with their group and suggest that the spontaneous tendency to synchronize with others is diminished in individuals with high ASD traits, though it is recovered when synchronization is intentional.

## Introduction

In the animal kingdom, birds and mammals protect their offsprings by aligning their behaviors to discourage predators^1^: penguins dive in groups to enhance their ability to catch fish^2^; fireflies use synchronous flashing behavior as part of their mating ritual^3^; and joint affect levels within a group facilitate the pursuit of common goals^4^. In humans, multi-person interactions that involve herding occur daily, whether singing in a choir, clapping at a theatrical performance or cheering at the football field. The prevalence of herding across the animal kingdom suggests that this behavior may promote survival. Indeed, herding provides major advantages^1,5^ ranging from promotion of foraging efficiency^6,7^ and reduction of predation risk^8^ in animals, to enhancing a group’s sense of unity, solidarity^9–11^ and emotional wellbeing^12^ in humans.

**Figure 1:**
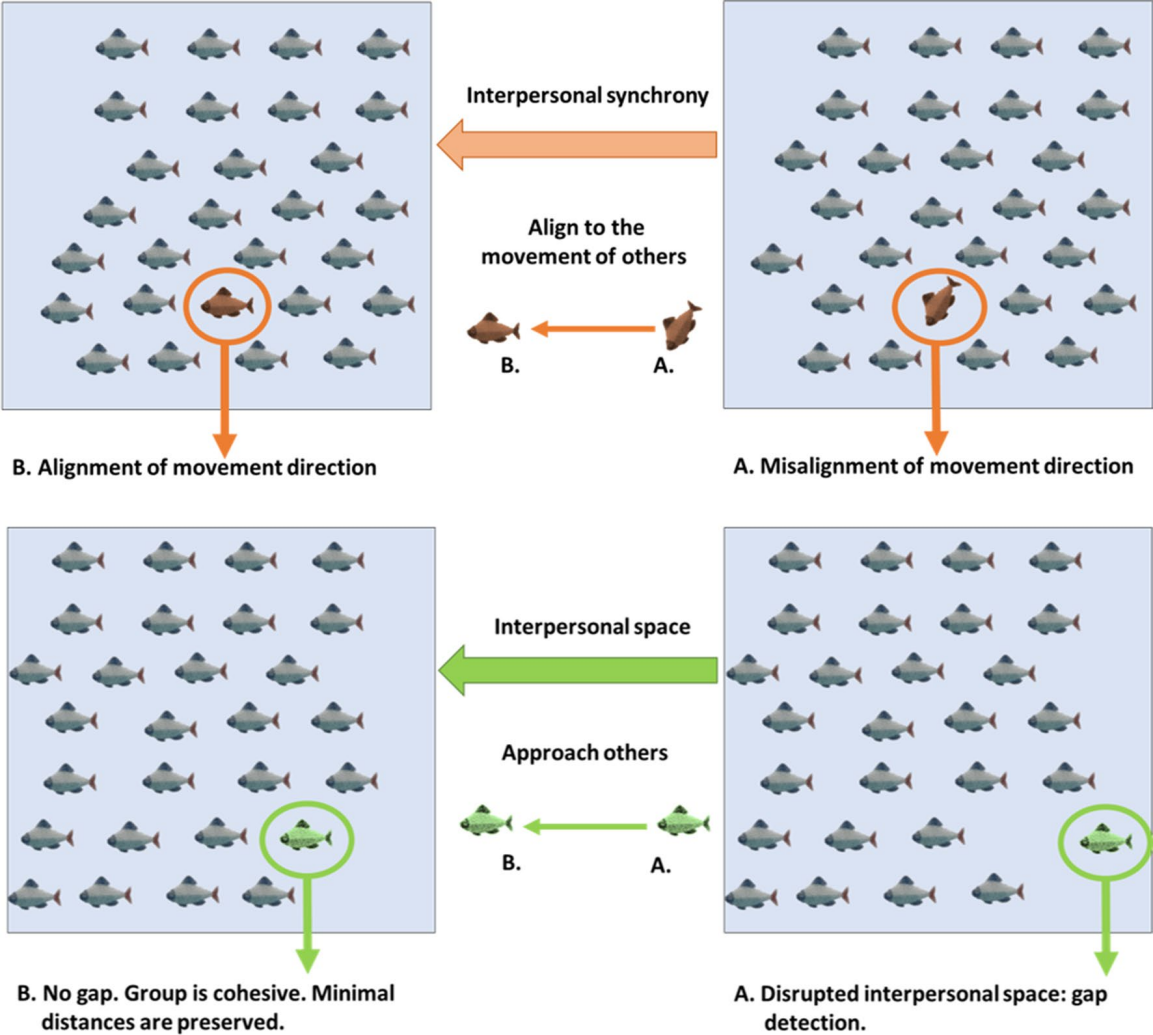
Principles of collective Herding across species. According to this model, herding entails synchronizing the direction of one’s movement with the movement of others, approaching them to maintain greater cohesion while keeping sufficient separation (minimal distance).

The regularity of various forms of herding in animals and humans may imply the existence of underlying principles that govern these behavioral forms. A well-accepted model of herding argues that there are three basic principles that guide herds: schools of fish, flock of birds and mammals. These principles include aligning with the direction of movement of those that are close by, and at the same time maintaining group cohesion by approaching other individuals in the herd, while separation is indicated by keeping distance to avoid intrusion^13–15^. These principles indicate that the mechanisms that underlie herding involve two systems: a system of interpersonal synchrony that allows alignment with other individuals and a system regulating interpersonal space through approach and avoidance from other individuals in a group (Fig. 1). Previous studies in humans examined herding mainly by examining interpersonal synchronization, from oscillatory limb and body motion synchronization^16–19^ ,synchronized group dancing^20^ and synchronized hart rate during collective rituals^21^. Despite initial evidence that humans tend to herd^15^, the spontaneous emergence of unstructured herding in human groups, involving increased interpersonal synchrony and decreased interpersonal space, is yet to be established. Therefore, the first aim of the current study was to examine, in a virtual environment, whether humans spontaneously herd within a group.

Human synchronization, the mechanism that facilitates movement alignment through interpersonal synchrony, is defined as the mutual, dynamic and simultaneous temporal alignment of individuals’ actions in order to attain behavioral similarity^22^. Interpersonal synchrony can be divided into two complementary processes: intentional and spontaneous^23,24^. Spontaneous synchronization refers to synchrony that emerges unconsciously, as apparent in rhythmic actions and body postures, when there is no explicit social goal of synchronization. Examples include the in-phase clapping of an audience^25^, unintentional synchronous walking^26^, limb alignment^27^ and implicit vocal^28^ and facial expression syncronization^29^. Intentional synchronization, in contrast, includes conscious and intended synchronous presentations involving planned movement alignment, such as dance routines, coordination in musical ensembles, marching soldiers and collective rituals^30^. Previous studies found that individual differences and dispositional personality tendencies can mediate both intentional^24^ and spontaneous^31–33^ synchrony with others, thus suggesting a synchrony performance range in which individuals may vary. Though intentional and spontaneous synchrony share considerable similarities (e.g., simultaneous alignment between individuals), these two abilities differ in the extent of awareness of others’ actions and in the level of monitoring required. Intentional synchrony involves elevated awareness of others’ actions while actively attempting to align with others, whereas spontaneous synchrony emerges unconsciously and involves low efforts to adjust and minimal awareness of others’ actions^26^. Due to the reduced demand for awareness, an individual’s spontaneous synchrony performance is not necessarily bound to his/her intentional performance. Indeed, this dissociation has been demonstrated by previous studies that found differences in individuals’ intentional synchrony performance but not in their spontaneous synchrony performance^34^ and vice versa^35^.

The second system presumably involved in herding involves interpersonal space regulation which regulates approaching toward the group in order to achieve cohesion, while maintaining separation from others by keeping a certain personal space to avoid intrusion. Interpersonal Space is the physical distance between interactive bodies present at the same time and place. The space kept between humans has social meaning as it sets the spatial boundaries of the interaction^36^. The Interpersonal Space system has a substantial part in shaping the interaction’s properties since it affects the level of intimacy that is comfortable, appropriate and safe^37^. Interpersonal Space is fundamental to all types of social interactions and can be observed and measured by the proximity between individuals. It varies between species^38^, status^39^ and cultures^40^. Much like the observed variance seen in synchrony performance, interpersonal space is also subjected to individuals’ preference, reflecting their personality differences^37,41–43^. Moreover, similarly to the disparities seen between intentional and spontaneous synchrony abilities, the interpersonal space between people is influenced by the properties of the interaction as well^44,45^.

Although interpersonal synchronization and space regulation are regarded as components of the mechanism of herding, these two systems had been studied separately, mainly in dyadic settings. Nevertheless, real-life human social behaviors are complex and not restricted merely to dyadic interaction. In fact, many of our day-to-day social encounters involve interacting with more than one individual at a time, thus, stressing the high value of “going social” by examining real-time interaction of real groups to increase ecological validity^46^.

Herding requires each group member to recognize and anticipate not only the actions of one other member, but rather the entire group’s continuously changing trajectory. Thus, herding requires simultaneously paying attention to numerous social cues from different origins while accounting for their distinct saliency and dependency^47^. Hence, investigating herding requires examining group dynamics by assessing the behavior of an individual simultaneously interacting with multiple partners. Indeed, a previous study showed that coordination levels in humans are affected by the specific group structure and the interconnection between group members^48^. Other studies also suggested that group size affects the dimensions of alignment response^49^, stressing the notion that some phenomena that can occur in groups cannot occur in pairs (e.g., communication structures, subgroup formation). Therefore, investigating the characteristics of herding in human groups may lay the groundwork for understanding group dynamics.

Although herding may occur naturally in groups, under certain conditions some degree of impairment leads to reduced tendency to herd with the group. Autism Spectrum Disorder (ASD) is one of a growing category of neurodevelopmental disorders marked by potential dysfunction in herding^50^. Among the diagnostic criteria for ASD are deficient reciprocity during social interaction, impairments in nonverbal communication and difficulty in adjusting to social context^51^. With regard to interpersonal synchrony in ASD, previous studies have noted that individuals with ASD exhibit impairments in interpersonal synchrony ability^52,53^, though, conflicting results indicate intact synchronization^54^. Likewise, the distinction between spontaneous and intentional interpersonal synchrony in ASD bears contradicting findings. While it has been argued that individuals with ASD show impairments in both intentional and spontaneous interpersonal synchrony^52^, there is evidence suggesting that individuals with ASD have a specific deficit in spontaneous synchronization^55^, presumably since it is not goal-directed. Contradictory findings are also reported with respect to ASD and interpersonal space. While some studies report individuals with ASD display a tendency to maintain greater and inflexible interpersonal space^56^, others point to a reduced personal space in ASD^57^. A possible unifying framework suggests that individuals with ASD show high variance in interpersonal distance preferences, choosing either interpersonal distance that is too close or too far from others^58^.

Note that the bulk of research on ASD to date focused on social interaction within dyads, thus limiting our ability to understand complex social behavior in groups. Therefore, the question of whether individuals with ASD or ASD traits exhibit impaired herding during genuine group interaction remains unanswered. Here, we were interested in examining whether the tendency to intentionally and spontaneously herd within a group is associated with the level of ASD traits of the individual group members.

**Figure 2:**
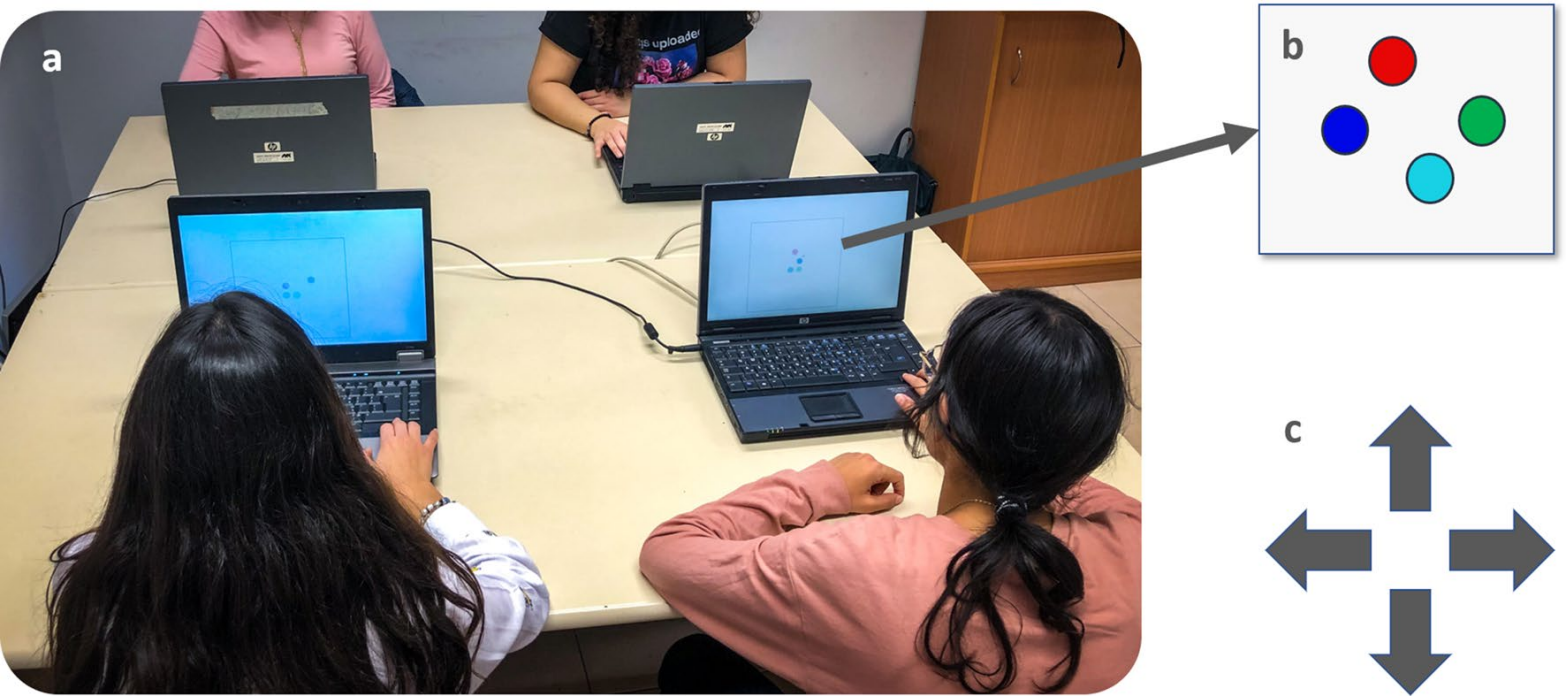
Task set-up, visual display and stimuli. (**a**) four participants sit together, each facing a personal laptop displaying the same visual image. (**b**) Visual display of the task stimuli: each participant is assigned a different color circle (red, blue, green or turquoise). (**c**) Task stimuli performance: each player controls the circle's movement using the four arrow keys and their combinations (creating additional diagonals). For a detailed description please see the “Methods”. For a video presentation please visit our website: https://sites.google.com/edu.haifa.ac.il/sans/lab-tasks?authuser=0.

**Figure 3:**
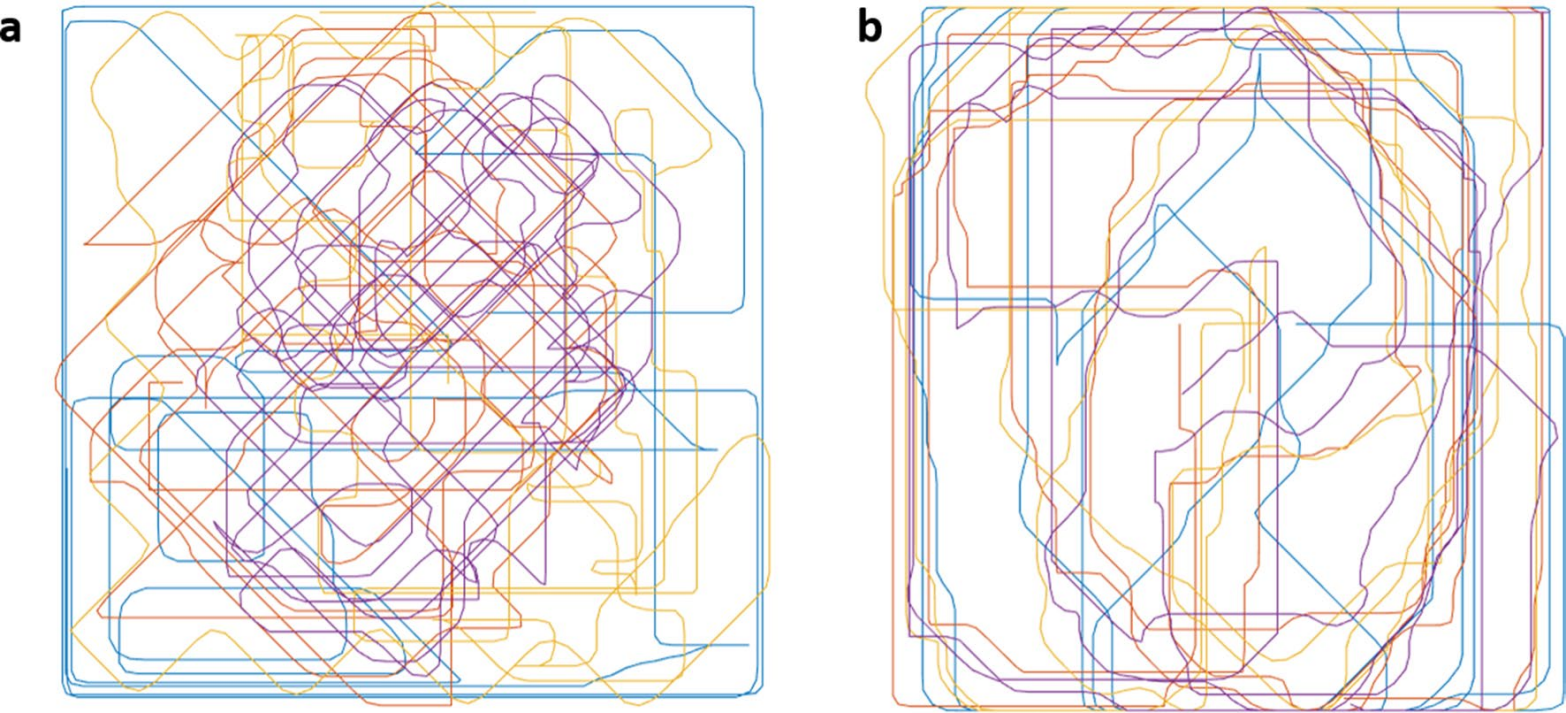
(**a**,**b**) Graphical presentation of a specific spatial pattern of movement of a single foursome for the entire session (3 min × 2 conditions). The different colors represent the different participants in the two condition (e.g., SH, IH). (**a**) Spatial pattern during spontaneous herding condition and (**b**) spatial pattern during intentional herding condition.

The purpose of the present study was to address these issues in a controlled setting with real social interactions. To this aim, we had four participants sit together at the same square table in a room, each facing a computer screen assigned to him/her (Fig. 2a). To assess herding we developed a computer-based multi-agent paradigm in which four participants were represented on the computer screen as different colored circle-shaped figures (Fig. 2b). By using different clients connected via a closed network, we facilitated a real-time interaction between the participants as they moved their circles on the computer screen (see “Methods”, “Herding task setup”, for details). The participants were instructed to use the keyboard arrows to control the movement of their colored circle-shaped figures (Fig. 2c). Keyboard usage was chosen since it is a very simple and familiar interface that facilitates the investigation of human motion which spans across usage of different modalities, organs and interfaces^59^. We then tracked the group’s movement trajectory under two conditions: spontaneous herding (SH) (Fig. 3a) and intentional herding (IH) (Fig. 3b), and calculated herding performance (i.e. directional synchrony, cohesion and separation) for each condition (see “Methods”, Measuring herding, for details). Prior to performing the herding task, participants completed the autism quotient (AQ) test^60^ to determine their level of ASD traits (see “Methods”, “The autism quotient (AQ) scale”, for details).

The movement patterns of each individual within the group were recorded throughout the entire session (two conditions; SH, IH, each lasting three minutes and divided for analysis purpose into six segments of 30 s). Next, we generated a simulated control trial in which participants were reassigned into pseudo groups by a random reshuffling of their movement patterns. Thus, for each participant we had two types of assignments: a real assignment (assignment to the group the participant had interacted with) and a pseudo assignment (assignment to a pseudo group). We compared participants’ herding in the real assignment to their performance in the pseudo assignment. This comparison enabled us to examine the emergence of herding in a real setting as opposed to a pseudo setting in which human interaction in essence did not take place. Thus, we compared directional synchrony, cohesion and separation in the task’s two conditions (SH, IH) during real social interaction (real) to the performance during pseudo (control) interaction (see “Methods”, “Statistical analysis” for details).

Similarly, to examine whether individuals with high ASD traits exhibit distinct herding tendencies we compared the participants’ herding (i.e. directional synchrony, cohesion and separation) of individuals with high ASD traits to the performance of individuals with low ASD traits in both task conditions. Since previous findings point to major variations in ASD-related traits among healthy individuals as well as across the entire population^61^, we sought to examine herding specifically in neurotypical individuals, similar to Neufeld et al.’s work^62^. Examining neurotypical individuals allows addressing quantitative differences in ASD symptom severity (by measuring ASD traits) without the need to account for additional variables that can influence performance, such as intelligence range^63^, motor impairments^64^ and comorbid disorders^65^.

## Methods

### Ethics statement

The Institutional Review Board (IRB) at the University of Haifa approved the experiment, including the written consent procedure. All participants provided their written informed consent to participate in the study. All methods were carried out in accordance with relevant guidelines and regulations.

### Participants

Our participants were 136 healthy University of Haifa undergraduates (105 women and 18 men (mean age 22.24 ± 2.9) who participated for class credit or payment. No gender differences in task performance (F =  − 0.07, SD = 0.87, M = 0.108, SD = 0.87, ns) or AQ score (F = 17.1, SD = 4.85, M = 16.33, SD = 5.06, ns) were observed, possibly due to the smaller number of men included in the study. Participants were assigned to 34 foursomes. Thirteen participants (including three foursomes and one individual participant) were excluded either for not completing the required questionnaires or for not completing the task. All participants had normal or corrected-to-normal vision. Exclusion criteria included any history of neurological or psychiatric condition or chronic illness. All participants were unaware of the experimental hypothesis.

### Procedure

In every session, all four participants sat together at the same square table in a room, each facing a computer screen assigned to him/her (Fig. 2). Prior to performing the herding task, participants completed the autism quotient (AQ) test^60^ to determine their level of ASD traits.

The Autism Quotient (AQ) scale^60^ is a self-administered instrument for quantifying the position of any given individual on a continuum ranging from ASD to neurotypical. The scale provides a score that measures the degree to which an adult with normal intelligence has traits associated with autistic spectrum disorder. It includes 50 statements divided into five subcategories relevant to autism (social skills, attention switching, communication, imagination and attention to detail). For each statement, the participant chooses one of four responses: definitely agree, slightly agree, slightly disagree or definitely disagree. The scale’s scores range from zero to 50, and a high score indicates a high level of autistic traits. The AQ scale is a highly reliable measure for quantifying ASD traits with strong test–retest reliability, high internal consistency and very good discrimination quality between healthy individuals and individuals with ASD^60^. Based on the tool’s guidelines, a score of 32+ is the cutoff for distinguishing individuals with clinically significant levels of autistic traits.

#### Assessing ASD traits

Each participant’s total score on the AQ scale as well as all the scores on the subscales (social skills, attention switching, communication, imagination and attention to detail) were calculated according to self-reported response. The mean AQ total score (M = 16.98, SD = 4.85) and all the subscale scores (M = 2.12, M = 4.45, M = 1.98, M = 2.46, M = 5.97) were in-line with the reported AQ distribution in the normal population according to the instrument’s description^60^ (AQ total score range: 22, ranging from 7 to 29, with a variance of 23.54). For the purpose of the analysis, we divided our participants into two groups of high (N = 53) and low (N = 70) ASD traits according to the median AQ score in our sample (med = 17), which corresponds with the mean AQ score in the general student population (mean = 17.6)^60^.

#### Herding task setup

Four client computers were connected to a server via a dedicated network. Each client handled the input and output for one of the participants. The participant controlled a moving circle using the four arrow keys, and the movement of the four circles (of different colors), controlled by the four participants, was presented on each of the screens (screen size: 14.10″; screen resolution: 1280 × 800 WXGA; size of the arena: width—11.20″, height—8.40″). The duration of each condition was 3 min. The speed of movement of each circle was determined by the vector sum of movement in the four major directions, each of which was proportional to the duration for which each respective key was pressed. Communication between each client and the server was executed asynchronously at about 5 Hz and post-processing interpolation of all data was conducted at a rate of 5 Hz, such that data for all participants shared matching sample times. At the beginning of the task, four colored circles appeared on all four screens: red, blue, green or turquoise (see Fig. 2a). Each colored circle was assigned to one of the four players. Participants were instructed not to talk to or touch each other during the experiment.

The task consisted of two conditions, each lasting 3 min (180 s): a spontaneous herding condition (SH) and an intentional herding (IH) condition. Each condition was later divided into six segments of equal duration (30 s) for the purpose of the analysis. In the SH condition, participants were instructed to move their circles freely by means of the four keyboard arrows and their combinations (additional four diagonals, see Fig. 2b). In the IH condition, participants were instructed to move the circles using the keyboard arrows as before, only this time they were explicitly asked to synchronize with each other as a group (see Fig. 3). Participants completed the spontaneous herding condition first and then the intentional herding condition. This order was maintained for all participants based on a preliminary pilot study carried out in our lab showing that instructed synchrony may affect the emergence of spontaneous herding.

#### Pseudo groups

To simulate a control trial in which participants did not interact with one another, we created “pseudo groups” by reshuffling the participant’s movement patterns and assigning them randomly into new groups of four. Each pseudo group consisted of four participants, each from a different real group. Thus, we could calculate two sets of measures for each participant; one based on the real group the participant had performed the task with, and one based on the "pseudo group" the participant had been assigned to.

#### Herding measurements

To assess herding, we calculated three measures, each corresponding to one of the three aforementioned principles presumed to guide herds (see “Introduction”) and relating to one of the two systems of herding (i.e. interpersonal synchrony and interpersonal space). The alignment in movement direction (directional synchrony) was used as a measure for interpersonal synchrony. Cohesion and separation were calculated as two measures of interpersonal space.

*Alignment* was assessed by examining the directional correlation between the movement of a specific participant and the movements of her/his group throughout the task. Directional correlation^47^ is the cosine of the angle between the velocities of each pair of players . The directional correlation between the i participant and the j participant is given by $\frac{<{v}_{i},{v}_{j}>}{|{v}_{i}||{v}_{j}|}$, where <v_i_v_j_> is the inner product of the velocities of the two participants, and |v_i_| and |v_j_| are the magnitudes of the velocities of the *i* participant and the *j* participant, respectively. Higher correlation indicates stronger tendency for herding. As mentioned above, data were collected at a sampling frequency of 5 Hz. For each condition (SH, IH), we obtained the velocity of each player at each time point and then calculated the directional correlation. For analysis purposes, we divided the data (900 time points per game) into six segments of equal duration (150 time points per segment). Directional correlations between each participant and each of the other participants were calculated for lags varying from − 2  to + 2 s, in 0.2-s increments (a total of 21 correlations for each pair of participants at each sample). The average correlation for each pair of participants at each lag across the duration of each segment was then calculated (21 averages per pair in each segment). The maximal correlations (i.e., the maximal values among these 21 averages), and the corresponding lags, in seconds, were then averaged across all pairs formed by a given participant and each of his/her group members to yield the mean of maximal correlation, and the corresponding lag, for that participant in each of the segments. Examination of the maximal directional correlation suggested the highest correlation between participants at zero-lag. Results of single-sample t-tests indicated that the averages of these lags across participants did not differ from zero in any of the segments. For more information see supplementary analyses (Supplementary Table S1). Finally, since directional synchrony is represented by correlations, Fisher's z-transformation was applied to these means to normalize their distribution^66^. Tests of normality were then performed separately for the mean zero-lag correlations for the two conditions. The results indicated an absolute skew value of 1.72 and 0.051 and an absolute kurtosis of 6.02 and − 0.24 for the SH and IH conditions respectively, allowing for the assumption of normal distribution^67^.

*Cohesion* was assessed using the distance (cm) from a shared center of mass. Smaller distances between the participant and the shared center of mass indicates a stronger tendency for herding (higher scores suggest decreased tendency for herding). For each condition (SH, IH), we obtained the distance of the participant from a shared center of mass of all other group members at each time point (total of six segments, 900 time points per condition at a sampling rate of 5 Hz), and calculated the average distance for each 30-s segment. Corresponding with the previous reported measure, cohesion was calculated for each segment at zero-lag.

*Separation* was examined by determining the minimal distance (cm) each participant maintained from the other participants during each 30-s segment. Smaller distances between the participant and other group members indicates less separation between individuals, hence, stronger tendency for herding. The minimal distance was again calculated in each segment at zero-lag (total of six segments, 900 time points per condition at a sampling rate of 5 Hz).

To examine the relationships between the three measures we created according to the three principals of herding, we performed a linear correlation analysis between all three variables. The results indicated a significant negative correlations between the measure of interpersonal synchrony (directional synchrony) and the measures of interpersonal space (cohesion, separation), suggesting that as the directional synchrony between group member increases, interpersonal space diminishes [correlation between directional synchrony and cohesion: r_p_ = − 0.636, p = 0.000; correlation between directional synchrony and separation: r_p_ = − 0.371, p = 0.000]. Furthermore, a significant correlation was found between the two measures of interpersonal space (cohesion and separation) [r_p_ = 0.68, p = 0.000].

### Statistical analysis

All reported analyses were two-sided and conducted using IBM SPSS version 25.

### Herding in real vs. pseudo assignment

To examine whether participants exhibit differences in herding during real assignment as compared to pseudo assignment under the different conditions (e.g. SH, IH), we conducted a binary mixed-model analysis similar to Gvirts el al.^68^, with group assignment score as the dependent variable (0 = real assignment, 1 = pseudo assignment) and condition (0 = spontaneous herding, 1 = instructed herding) as a fixed effect. This analysis was repeated three time, once for each of the herding measures as an additional fixed effect. The group number (specifying foursome ID number) and the time segment (the number of the segments ranging from 1 to 6) were the random effects (Table 1). If herding is a naturally occurring phenomenon, we would expect this analysis to differentiate between real and pseudo interaction in both conditions. The sample included 123 participants, all engaged in SH and IH conditions. For each participant we obtained the measurements of directional synchrony, cohesion and separation across six-time segment in the two conditions for both the real and the pseudo assignment (6 × 2 × 2 = 24). Thus, the data set was constructed of N = 2952 (24 herding measurements × 123 participants = 2952).

**Table 1:** Binary mixed-model using herding measures to predict group assignment (real/pseudo)—fixed coefficients.

Model Term	Coefficient	Std. error	t	Sig	95% confidence interval		Exp (coefficient)	95% confidence interval for exp (coefficient)	
					Lower	Upper		Lower	Upper
Intercept	− 0.028	1.7200	− 0.016	0.987	− 3.400	3.345	0.973	0.033	28.360
Directional synchrony * [condition = IH]**	− 9.673	0.5443	− 17.771	0.000	− 10.740	− 8.606	6.295E−5	2.165E−5	0.000
Directional synchrony * [condition = SH]**	− 1.800	0.3738	− 4.815	0.000	− 2.533	− 1.067	0.165	0.079	0.344
Condition = IH**	2.486	0.1505	16.522	0.000	2.191	2.782	12.019	8.947	16.144
Condition = SH	0^b^	–	–	–	–	–	–	–	–
Directional synchrony	0^b^	–	–	–	–	–	–	–	–
Intercept	− .313	1.5547	− 0.202	0.840	− 3.362	2.735	0.731	0.035	15.412
Cohesion * [condition = IH]**	2.660	0.1359	19.573	0.000	2.393	2.926	14.294	10.950	18.658
Cohesion * [condition = SH]	0.068	0.0499	1.359	0.174	− 0.030	0.166	1.070	0.970	1.180
Condition = IH**	− 5.645	0.3563	− 15.846	0.000	− 6.344	− 4.947	0.004	0.002	0.007
Condition = SH	0^b^	–	–	–	–	–	–	–	–
Cohesion	0^b^	–	–	–	–	–	–	–	–
Intercept	0.443	1.7701	0.250	0.802	− 3.027	3.914	1.558	0.048	50.101
Separation * [condition = IH]**	2.184	0.1769	12.342	0.000	1.837	2.531	8.881	6.277	12.564
Separation * [condition = SH]**	− 0.809	0.0919	− 8.803	0.000	− 0.989	− 0.629	0.445	0.372	0.533
Condition = IH**	− 1.242	0.1105	− 11.243	0.000	− 1.459	− 1.026	0.289	0.232	0.359
Condition = SH	0^b^	–	–	–	–	–	–	–	–
Separation	0^b^	–	–	–	–	–	–	–	–

Probability distribution: Binomial.

Link function: Logit^a^.

^a^Target: group assignment (real/pseudo).

^b^This coefficient is set to zero because it is redundant.

**p < 0.001.

### Predicting level of ASD based on herding

To compare herding between individuals with high and low ASD traits, we conducted a binary mixed-model analysis, with AQ group type score as the dependent variable (0 = Low AQ, 1 = High AQ). The fixed effects were condition (0 = spontaneous herding, 1 = instructed herding) and herding measures. The group number (specifying foursome ID number) and the time segment (the number of the segments ranging from 1 to 6) were the random effects (Table 2). This analysis was repeated three time, once for each of the herding measures as an additional fixed effect. If individuals with low and high ASD traits exhibit differences in directional synchrony, cohesion or separation between the task conditions, we would expect this analysis to differentiate between the two groups. The sample included 123 participants, all engaged in SH and IH conditions. For each participant we obtained the measurements of directional synchrony, cohesion and separation across six-time segment in the two conditions (6 × 2 = 12). Thus, the data set was constructed of N = 1476 (12 herding measurements × 123 participants = 1476).

**Table 2:** Binary mixed model using herding measures to predict AQ group (high/low AQ)—fixed coefficients.

Model term	Coefficient	Std. error	t	Sig	95% confidence interval			Exp (coefficient)	95% confidence interval for exp (coefficient)	
					Lower		Upper		Lower	Upper
Intercept	− 0.243	1.3928	− 0.175	0.861	− 2.975		2.489	0.784	0.051	12.046
Directional synchrony * [condition = IH]	− 0.290	0.1991	− 1.459	0.145	− 0.681		0.100	0.748	0.506	1.105
Directional synchrony * [condition = SH]*	− 0.884	0.4290	− 2.060	0.040	− 1.725		− 0.042	0.413	0.178	.959
Condition = IH	0.169	0.1752	0.962	0.336	− 0.175		0.512	1.184	0.839	1.669
Condition = SH	0^b^	–	–	–	–		–	–	–	–
Directional synchrony	0^b^	–	–	–	–		–	–	–	–
Intercept	− 0.712	1.8521	− 0.385	0.701	− 4.345		2.921	0.490	0.013	18.553
Cohesion * [condition = IH]	0.168	0.1022	1.639	0.101	− 0.033		0.368	1.182	0.968	1.445
Cohesion * [condition = SH]*	0.131	0.0652	2.013	0.044	0.003		0.259	1.140	1.003	1.296
Condition = IH	0.221	0.2736	0.808	0.419	− 0.316		0.758	1.247	0.729	2.134
Condition = SH	0^b^	–	–	–	–		–	–	–	–
Directional synchrony	0^b^	–	–	–	–		–	–	–	–
Intercept	− 0.406	1.8423	− 0.221	0.825	− 0.402		3.207	0.666	0.018	24.717
Separation * [condition = IH]	0.285	0.2199	1.298	0.195	− 0.146		0.717	1.330	0.864	2.048
Separation * [condition = SH]	0.148	0.0872	1.692	0.091	− 0.023		0.319	1.159	0.977	1.375
Condition = IH	0.062	0.1397	0.440	0.660	− 0.213		0.336	1.063	0.808	1.399
Condition = SH	0^b^	–	–	–	–		–	–	–	–
Directional synchrony	0^b^	–	–	–	–		–	–	–	–

Probability distribution: Binomial.

Link function: Logit^a^.

^a^Target: AQ group type.

^b^This coefficient is set to zero because it is redundant.

*p < 0.05.

### The effect of ASD traits on herding

To examine the effects of AQ score as a continuous variable and experimental condition (SH, IH) on each of the herding measures (i.e. directional synchrony, cohesion, separation) linear mixed model (LMM) analyses were conducted. Since in each of the experiment’s trials participants were randomly assigned into groups of four, our sample contains clustered data such that the performance of each participant in a specific foursome is dependent upon the performance of all other participants in that group. Moreover, each foursome included a distinct composition of participants’ characteristics (e.g. distinct AQ score, degree of acquaintance, gender etc.) that may affect their performance in the task. To account for this random effect we used Linear Mixed Model (LMM) analysis^69^. The LMM approach offers many additional advantages over traditional repeated-measures ANOVA including the ability to model correctly group data by handling random effects. Here this approach was used to deal with the issue of interdependence between the scores within each group. By using LMM analysis we were able to include cluster-level factors such as condition type (SH, IH), while adjusting for the random effects associated with each clustered foursome. In addition, to account specifically for the possible effect of previous acquaintance between participants we conducted a linear correlation between the degree of acquaintance (participants self-report on a Likert scale between 1 and 10) and all of the herding measures.

The relationship between each of the herding measures in the different conditions and the level of ASD traits as a continuous variable were examined by a LMM analysis with all three herding measures as the dependent variables, group number (specifying foursome ID number) as random effect, condition (SH, IH) and time-segments (the number of the segments ranging from 1 to 6) as a within-subject factor and AQ score as a between-subject covariate.

## Results

### Herding in real vs. pseudo assignment

The binary mixed model analyses revealed that all of the herding measures predicted the dependent variable group assignment (real/pseudo) during the intentional herding condition [Table 1; directional synchrony × condition = IH: t = − 17.771, p = 0.00; cohesion × condition = IH: t = 19.573, p = 0.000; separation × condition = IH: t = 12.342, p = 0.000]. Thus, during intentional herding the odds of belonging to the pseudo group were significantly lower when directional synchrony increased [Table 1; odd ratio = 6.295E−5] or when the measures of cohesion and separation increased (participants showed greater distance to the shared center of mass and greater distances from other) [Table 1; odd ratio = 14.294 and 8.881, respectively].

In the spontaneous herding condition, the binary mixed-model analyses suggest that both directional synchrony and separation predicted group assignment (real/pseudo) [directional synchrony × condition = SH: t = − 4.815, p = 0.001; separation × condition = IH: t = 12.342, p = 0.000]; whereas the cohesion measure did not differentiate between the real and pseudo assignment [cohesion × condition = IH: t = 1.359, P = 0.174]. As with the intentional herding condition, the odds of belonging to the pseudo group were significantly lower as directional synchrony increased [Table 1; odd ratio = 0.165]. However, contrary to the intentional herding condition, the odds of belonging to the pseudo group were significantly lower as the separation between individuals decreased [Table 1; odd ratio = 0.445].

Taken together, these findings indicate that participants who are engaged in real interactions exhibit greater interpersonal synchrony during both types of herding (intentional and spontaneous). Moreover, in accordance with the core principles of herding, participant who are engaged in real interactions during intentional herding, exhibited diminished interpersonal space (smaller distance to the shared center of mass and smaller distances from other) than the pseudo groups. In contrast, participant who are engaged in real interactions during spontaneous herding exhibited greater separation (greater distances from each other) than the pseudo groups. The measure of cohesion did not differentiate between real and pseudo groups during the spontaneous herding condition.

**Figure 4:**
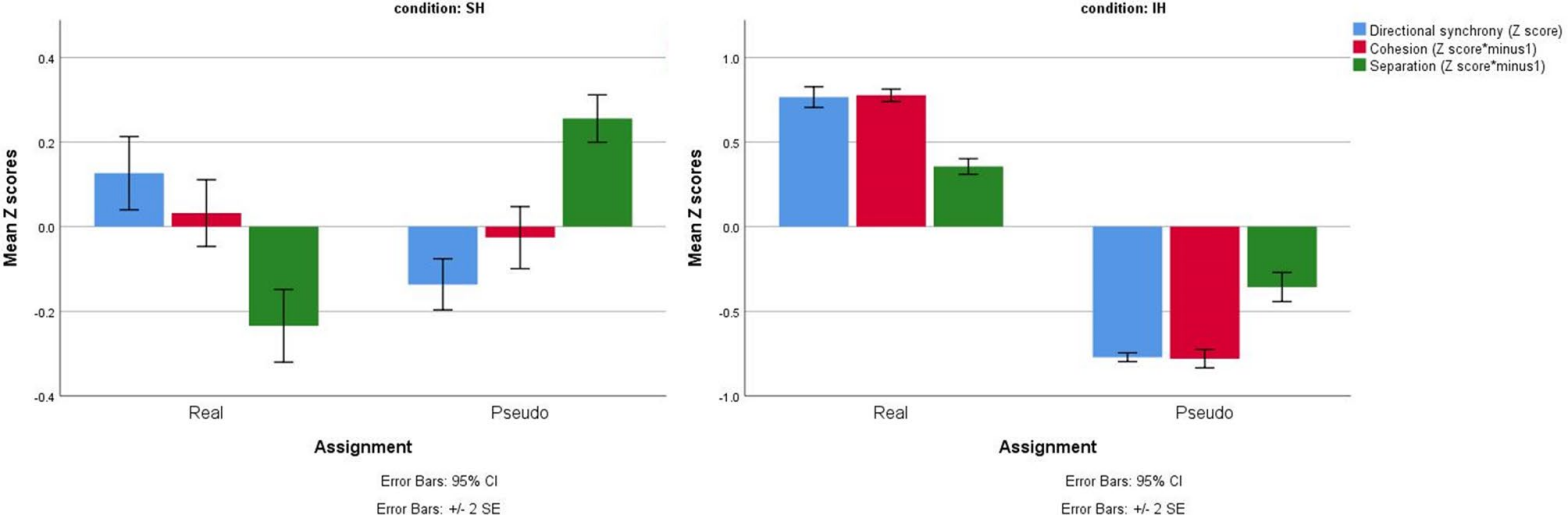
Directional synchrony, cohesion and separation Z scores of participants assigned either to real or to pseudo groups in the task’s conditions (SH,IH). Clustered Bar Mean of Z scores of all three measures of herding: Z_Directional synchrony_ and Z_cohesion_ Z_separation_ (both multiplied by minus one) by assignment (real/pseudo). Higher Z scores indicate increased herding (greater interpersonal synchrony and diminished interpersonal space).

To follow the performance dynamics, visual presentation of all three measures in both real and pseudo assignment in each condition is provided (mean Z scores for the entire task’s conditions, Fig. 4). It should be noted that the Z scores of cohesion and separation were multiplied by minus 1, hence, higher Z scores in all three measures indicate increased herding, as higher Z scores represent greater interpersonal synchrony and diminished interpersonal space. The results suggest higher directional synchrony during real assignment than during pseudo assignment [real assignment: mean(sd) = 0.476(1.17)); pseudo assignment: mean(sd) = − 0.494(0.368)]. Similarly, the cohesion Z score in the pseudo groups in smaller compared to the real group [real assignment: mean(sd) = 0.421(1.02)); pseudo assignment: mean(sd) = − 0.439(0.761)], suggesting greater distances from the shared center of mass in the pseudo assignment. Finally, the separation Z score in the pseudo groups was slightly smaller than the Z score of the real group (real assignment: mean(sd) = 0.011 (1.1); pseudo assignment: mean(sd) = − 0.012 (0.88)), suggesting greater minimal distances between participants during pseudo assignment.

### Predicting herding based on ASD traits

**Figure 5:**
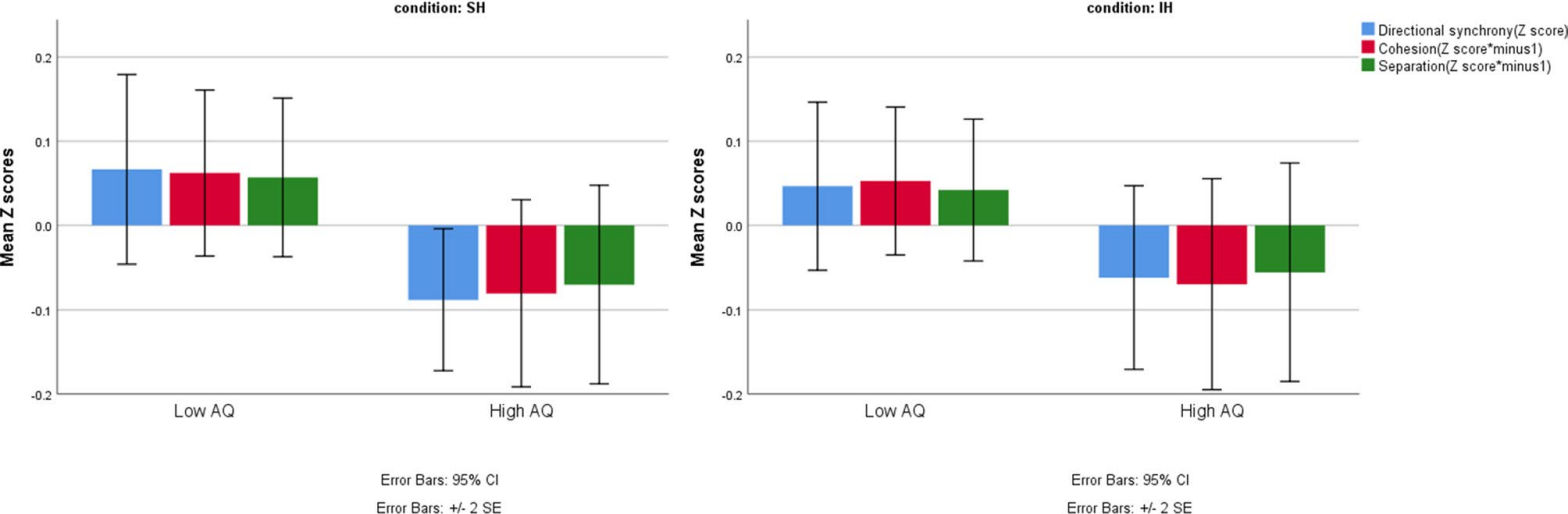
Z scores of all herding measures in spontaneous and intentional herding conditions. Directional synchrony, cohesion and separation Z scores of individuals with low and high AQ scores in the SH and IH conditions are presented separately.

The binary mixed model analyses revealed that directional synchrony and cohesion predicted the dependent variable AQ group (low/high) during the spontaneous herding condition (Table 2; directional synchrony × condition = SH: t = − 2.060, p = 0.04; cohesion × condition = SH: t = 2.013, p = 0.044;). Likewise, separation predicted the dependent variable AQ group (low/high) during spontaneous herding condition, though this effect was only marginally significant [separation × condition = SH: t = 1.692, P = 0.091]. During spontaneous herding, the odds of belonging to the high AQ group were significantly lower when directional synchrony increased [Table 2; odd ratio = 0.413], and significantly higher when interpersonal space increased (when the measures of cohesion and separation increased). Note that higher scores in these measures indicate lower levels of herding [Table 2; odd ratio = 1.14 and 1.159, respectively]. In contrast, the herding measures did not predict the dependent variable AQ group (low/high) during the intentional herding condition [Table 2; directional synchrony × condition = IH: t = − 1.459, p = 0.145; cohesion × condition = IH: t = 1.64, p = 0.101; separation × condition = IH: t = 1.298, p = 0.195;]. Inspection of the means revealed higher directional synchrony for individuals with low AQ than for those with high AQ only during the SH condition (low AQ mean(sd) = 0.055(0.209), high AQ mean(sd) = 0.0277(0.135)), but not during the IH condition (low AQ mean(sd) = 0.72(0.382), high AQ mean(sd) = 0.680(0.364)). Similarly, individuals with low AQ displayed decreased interpersonal space (decrease in the measures of cohesion and separation, thus, smaller distances from the center of shared mass and smaller minimal distances) than those seen for the high AQ group during the SH condition (cohesion: low AQ mean(sd) = 3.23(1.15), high AQ mean(sd) = 3.4(1.128); separation: low AQ mean(sd) = 0.82(0.82), high AQ mean(sd) = 0.92(0.89)). The performance dynamics can be seen in the visual presentation of herding measures of participants with high and low AQ traits during the different conditions (means of directional synchrony, cohesion and separation during SH and IH, Fig. 5).

### The effect of ASD traits on herding

**Figure 6:**
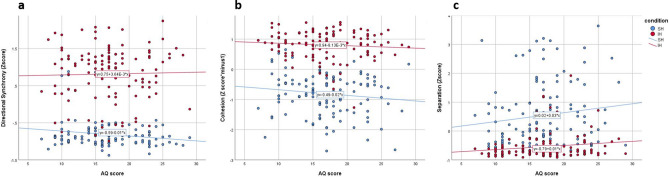
Linear correlations between individuals’ AQ score and the different measures of herding (Z scores). (**a**) A negative correlation between AQ score and directional synchrony is evident in the SH condition, but not in the IH condition, suggesting decreased directional synchrony as the AQ score increases only during spontaneous herding. (**b**) A negative correlation between AQ score and cohesion in both conditions, suggesting decreased cohesion as the individual AQ score increases. (**c**) A positive correlation between AQ score and separation in both conditions, suggesting as the individual AQ score increases so does the minimal distance between individuals (separation).

To examine the relationship between ASD traits and herding, a linear mixed model analysis (see “Methods”, “Statistical analysis”, for details), with directional synchrony as the dependent variable, group number as a random effect, condition (SH, IH) and time-segment as a within-subject factors and AQ score as a between-subject covariate was performed. Results revealed a significant effect for group number, indicating that the different composition of the group significantly influenced the directional synchrony among participants within the groups [F_(1,180.97)_ = 78.88, p = 0.000]. The main effect of AQ score was not significant [F_(1,180.97)_ = 0.77, p = 0.38], suggesting that overall AQ scores do not influence directional synchrony in general. However, the results revealed a significant interaction between AQ score and condition [F_(1,775.08)_ = 3.91, p = 0.048], suggesting that the directional synchrony is affected by ASD traits differently in each of the conditions (SH, IH) . In order to examine the source of the interaction effect, a follow-up analysis was conducted separately for each condition (SH, IH). Linear mixed model analyses were performed for each of the conditions (SH, IH), with directional synchrony as the dependent variable, group number as a random effects, time segment as a within-subject fixed effect and AQ score as a between-subject covariate. Results revealed a significant effect for AQ score in the SH condition [F(1,116.93) = 4.2, p = 0.043], whereas no effect was found for AQ score in the IH condition [F(1,118.85) = 0.116, p = 0.735] (Fig. 6).

To examine cohesion, a linear mixed model analysis was performed, with cohesion as the dependent variable, group number as a random effect, condition (SH, IH) and time segment as within-subject factors and AQ score as a between-subject covariate (see “Methods”). Here, again, results revealed a significant effect for group number, suggesting that the composition of the group influenced the degree of cohesion [F_(1,140.06)_ = 131.73, p = 0.000]. The interaction between AQ score and condition was not significant [F_(1,1874.06)_ = 2.23, p = 0.135]. On the other hand, a marginally significant main effect for AQ score was found [F_(1,140.06)_ = 3.09, p = 0.081], suggesting a possible effect of ASD traits across conditions wherein, as the participant’s’ AQ score increases, the distance from the shared center of mass is also increased (Fig. 6).

Similarly, to examine effects of ASD traits on separation, a linear mixed model analysis with separation (minimal distance from others) as the dependent variable, group number as a random effect, condition (SH, IH) and time-segment as a within-subject factors and AQ score as a between-subject covariate was performed (see “Methods”). Results revealed a significant effect for group number [F(1,183.8) = 14.76, p = 0.000]. As in the case of cohesion, the interaction between AQ score and condition was not significant [F(1,691.7) = 2.51, p = 0.114]. However, the analysis revealed a significant main effect for ASD traits across conditions [F(1,183.8) = 4.22, p = 0.041], suggesting an effect of ASD traits across the task’s conditions, wherein, as the AQ score increases, the minimal distance between participants increase as well (Fig. 6).

Finally, in order to examine possible effects of previous acquaintance between participants on herding, we conducted a linear correlation between the degree of acquaintance (the participants self-ratings on a Likert scale between 1 and 10) and each of the herding measures. No significant correlations were found between the degree of acquaintance and directional synchrony (r_p_ = − 0.086, p = 0.181) or cohesion (r_p_ = − 0.057, p = 0.37). However, a significant correlation emerged between the degree of acquaintance and separation (r_p_ = − 0.231, p = 0.000), indicating that as the degree of acquaintance increases, the minimal distances between the participants decreases.

## Discussion

The first aim of the current study was to examine, using a simple computerized task, whether humans engage in spontaneous herding behaviors as animal do. We developed a new paradigm in which both spontaneous and intentional herding could be assessed in human groups. Based on Fitt's law of stability of motor actions^59^, according to which the performance capacity of the motor system is relatively constant over a range of task conditions, it was reasoned that performance in a simple 2D movement task may represent complex human movement. Our second aim was to examine weather intentional and spontaneous herding is associated with ASD traits. Our novel approach views herding as a skill that varies among individuals and that may contribute to social behavior. Collective behavior in animals has been studied in both large groups^47^ and small groups^47^. Recent models of small group behavior suggest that groups of four mammals (rodents) exhibit complex movement patterns reflecting social interaction maps of group dynamics^70^. Our results are for groups composed of four participants, thus, revealing the mechanisms of herding in small groups.

The results showed that, similarly to group-living animals, humans tend to herd spontaneously with their group. Specifically, we found that even during a simple virtual social interaction, people are inclined to spontaneously synchronize their movement direction with their group members. The results reveal increased directional synchrony during both spontaneous and intentional social interaction compared to controls (assignment to random pseudo groups). These findings are in line with the well-established model of herding in animals^13,14^, suggesting that human groups share the same basic components of alignment with group-living animals, synchronizing their movement with no explicit intention to do so and with no apparent reward.

Notably, although during intentional herding participants indeed displayed diminished interpersonal space as expected, during spontaneous herding participants did not display the same effect. Thus, during spontaneous social interaction participants were not more cohesive compared to the pseudo groups and even showed increased separation from one another. These results might imply that humans’ herding is affected more by the enhanced alignment in movement direction than by the decrease in interpersonal space. Another explanation for the lack of diminished interpersonal space during spontaneous herding is the vast variance in interpersonal space preference^37,41–43^ that precluded us from finding a significant effect in this particular task, due to the task’s limitation of interpersonal space (the boundaries of the computer screen arena). Moreover, the greater distances between individuals, evident during spontaneous interaction, might reflect the participants need for preserving a comfortable distance from others, compared with the pseudo group in which no limitation of distance exist.

Previous studies demonstrated that collective synchronization emerges in various modalities, ranging from similar physiological arousal such as heart rate^21^ and respiratory rhythm^18^, to emotional contagion^71^ and verbal synchrony^72^, indicating that people tend to adapt their behaviors to those of the group. Due to the many manifestations of herding behavior, previous research stressed the importance of creating a unifying theoretical framework for characterizing herding in humans^73^. Herding has been widely studied in animal groups^14^, but far fewer studies have examined herding in humans. Although the evolutionary perspective assumes the existence of a shared mechanism for herding across species, there is limited evidence to support this claim^74^. Thus, the current study provides experimental support for the notion of a herd instinct in humans that adheres to the principle of alignment governing the animal realm.

Measuring herding and investigating its variations within individuals in different settings offers new avenues for examining essential social processes. As social beings, humans are embedded in social structures that guide their behavior by means of collective decisions^75^. Therefore, difficulties in herding may have wide-ranging implications for social interaction. ASD is a disorder known for impaired social skills and aberrant communication ability, as manifested in difficulties in adjusting and attending to others^76^. The existing literature on ASD focuses mainly on examining dyadic interactions, but little is known about the distinctive nature of social grouping in ASD and even less about herding in a group. Recent findings suggest that ASD traits exist in the general population, exhibiting the same etiology along a continuum with major variations^61^. Therefore, we sought to examine whether ASD traits (as measured by AQ score) influence an individual's ability to herd with a group. Our findings suggest a dissociation between the different elements of herding. Examination of interpersonal synchrony indicated that the level of ASD traits affects individual’s tendency toward spontaneously synchronizing with others’ movements. However, when explicitly asked to synchronize, individual’s performance did not differ between individuals with low- vs. high ASD traits. Thus, regardless of an individual's ASD traits, the ability to herd with a group can be recovered simply by making herding a goal-directed behavior. On the other hand, the degree of ASD traits affects individual’s tendencies to keep greater interpersonal space at all times, even when the task instructions imply being part of a group.

Support for the notion of aberrant spontaneous, but not intentional, interpersonal synchrony can be found in studies addressing the gap between spontaneous and intentional social skills among individuals with ASD. A review of studies conducted among high-functioning ASD individuals reported impairments in the spontaneous ability to mentalize and process information and a decreased tendency to spontaneously mimic others’ actions^35^. At the same time, these individuals demonstrated an intact capacity to attribute and process mental states explicitly and to imitate the same actions under explicit instruction. The dissociation between spontaneous and intentional social abilities could be the result of a weaker spontaneous attention to others’ actions in the ASD population^35^, and/or a diminished motivation towards social stimuli^77^. Evidence supporting these assumptions comes from studies of early development in individuals with ASD. Previous reports on 6-month-old toddlers later diagnosed with ASD indicated a diminished tendency to spontaneously attend to people and their activities, suggesting an early development bias that can possibly impact the emergence of social interaction patterns^78^. Additional support can be found in previous studies examining spontaneous social synchrony and oxytocin in ASD. Reports showed that both behavioral and neural effects of oxytocin are negatively correlated with ASD traits^79,80^. These findings along with the notion that oxytocin plays an important role in promoting interpersonal synchrony^81^ may point to a neuro-biological mechanism for the attenuation in spontaneous synchrony in individuals with high ASD traits.

Nevertheless, in the current study the dissociation between spontaneous and intentional skills emerged only partially when examining interpersonal space. The results indicated that alignment during the spontaneous herding condition but not during the intentional herding could predict the participants assignment into the high vs. low AQ group. However, we also found an overall tendency to keep greater distances from other players as a function of ASD traits, irrespective to the task’s conditions. The results also suggested that individual’s level of ASD traits predicted the tendency to be less cohesive with the group, although this effect was only marginally significant. These contradicting results are in line with previous findings suggesting an atypical regulation of interpersonal space in ASD^58^. While some studies reported increased interpersonal distances in ASD regardless of the situational characteritcs^56^, other studies suggest reduced interpersonal space in individuals with ASD^57^. Thus, it is possible that due to the large variation in interpersonal space preferences among participants, we could not detect a specific effect for ASD traits during spontaneous herding in interpersonal space measures of herding, but rather an overall effect of ASD traits. Another consideration for this inconsistency is the diverse sensory sensitivity in ASD that potentially affects interpersonal space preference across the entire spectrum of ASD traits^82^. It has been previously shown that individuals with ASD have difficulties in modulating sensory input, showing both over- and under-responsiveness to sensory stimuli^83^. Since sensory sensitivity levels have been shown to be predicted by interpersonal space preference, so that greater sensitivity is related to greater distance preference^43^, it could be that high variability in sensory sensitivity in our sample contributed to the lack of differences in interpersonal space measures during spontaneous and intentional herding. As the current study did not include a clinical sample, our results are limited to the neurotypical range. However, given the variation of ASD traits in the population^61^, we would expect to find similar patterns of behavior in a clinical ASD sample: diminished tendency to spontaneously synchronize with others together with intact ability to synchronize when explicitly asked. Indeed, previous studies in clinical samples of ASD^84^ as well as samples of typically developing individuals with high AQ scores^85^ support this assumption of impairments in implicit functions. Further support can be seen in studies reporting reduced neural functional connectivity during implicit learning in ASD^86^, suggesting altered neural adaptation during tasks that require implicit ability. More importantly, reports indicate that although individuals with ASD may have diminished implicit functions, they possess a normal ability to perform the same functions explicitly^87^. These results suggest that individuals with ASD may not be able to learn and understand subtle relationships implicitly as do typically developing (TD) individuals, though they do have the ability to compensate by learning and understanding a set of explicit rules. This conceptualization supports previous studies suggesting a new approach to understanding social impairments in ASD as a specified malfunction evident during real and complex social engagement^88,89^, thus, emphasizing the importance of characterizing social deficits in ASD^90^. A general dissociation between explicit and implicit human processes has been previously established. From an evolutionary perspective, researchers have argued that the unconscious and implicit induction systems were formed earlier and therefore involve patterns that are distinct from those of explicit mindful processes^91^. Empirical behavioral data support this dissociation, suggesting qualitative differences between implicit and explicit learning^92^. Additionally, recent neuroimaging studies demonstrate that distinct neural substrates are involved in conscious and nonconscious components of performance. For example, Destrebecqz et al.^93^ found that during learned sequence recollection, the striatum subtends the implicit component of performance, whereas the anterior cingulate/mesial prefrontal cortex (ACC/MPFC) subtends the explicit component. Though this dissociation is extensive, it appears to be prominent when addressing herding in ASD population. If so, a possible implication would be to invest efforts in addressing the gap between implicit and explicit herding in ASD by teaching individuals how to intentionally synchronize with their mates, since these are social gestures that are normally carried out spontaneously.

Although many studies on ASD highlight the negative consequences of social skill impairments exhibited by ASD individuals, diminished herding may have positive outcomes as well. Though herding is considered to represent a positive prosocial behavior, some have argued that it also bears negative ramifications, as seen in the earlier works of Sherif^94^ and Asch^95^. These studies stress the potential danger arising from herding by demonstrating how individuals are inclined to adhere to the group’s norms in the face of group pressure, even if these norms contradict their own opinions or are morally wrong. Additionally, previous studies suggest that individuals who are highly aligned with others are more constrained by the situation and therefore lack the freedom to explore and are less flexible in their ability to adapt. The results of these studies showed that weaker synchronization is associated with a more secure attachment style^96^ and predicts better problem-solving performance^97^. Thus, in some situations a lesser tendency to conform to the actions of others will not be considered as an impaired herding behavior, but rather will be thought of as firm individuality and a sense of freedom that may encourage creativity. Indeed, although previous study suggest that ASD individuals have poor imagination and creativity^98^, other studied indicate that these individuals demonstrate greater creativity^99,100^. Some researchers even contend that certain features of ASD, such as the ability to focus on a topic for very long periods of time, enhance creativity^101^. Therefore, in line with the assumption of a distinct and unique form of thinking among individuals with ASD, it is important to acknowledge a potential variation in spontaneous herding in the ASD population, though it is not necessary to amend this performance so as to align it with that of TD individuals.

The need to investigate the dynamics underling mutual coordination in humans is of high merit since herding is not created by an individual, but rather arises within the complexity of dynamic constrains^17^. Therefore, by examining solely the individual’s ability to align without relating to his/her group, we cannot provide holistic and valid examination of herding. The current study’s results suggest a possible effect for the specific composition of the group (e.g. participants’ AQ score, degree of acquaintance, gender etc.) on the participant’s tendency to herd with his/her designated group. Thus, not only the individual’s personal characteristics influence the tendency to herd, but also the assignment to a specific group with a unique set of qualities may affect the degree of herding. However, in the current study the groups varied in their compositions, which precluded a thorough analysis of the effects of composition in terms of AQ. Future studies should examine how the different compositions of AQ within the group affect the individual’s tendency to herd.

Lastly, the results indicated that the degree of acquaintance was positively correlated with the measurement of separation, but not with the other components of herding. Thus, it seems that previous familiarity with group members influenced the participants’ tendency to keep smaller distances from each other but did not affected their degree of interpersonal synchrony and cohesion. The current study’s limitation included a greater number of female participants compared to male participants that could have contributed to the lack of sex differences in AQ scores and task performance. Furthermore, we did not control the participant’s previous acquaintance. Future studies should address the issue of group composition by controlling variables such as gender, previous acquaintance and AQ compositions.

## Supplementary information

Supplementary Information.
