# Supplementary material for: Herding in human groups is related to high autistic traits
Source: Sci Rep. 2020 Oct 21;10:17957. doi: 10.1038/s41598-020-74951-8 (PMC7578000; doi:10.1038/s41598-020-74951-8)
Supplement: Supplementary file 1 — Supplementary Information. [file 41598_2020_74951_MOESM1_ESM.pdf]

Herding in Human Groups is Related to High Autistic Traits

Marton-Alper, I.Z.[1]*, Gvirts Provolovski, H. Z.[2], Nevat, M[1]., Karklinsky, M.[3], Shamay-Tsoory, S.G.[1,4]

[1] Department of Psychology, University of Haifa
[2] Department of Behavioral Sciences and Psychology, Ariel University
[3] Department of Computer Science and Applied Mathematics, Weizmann Institute of Science
[4] Integrated Brain and Behavior Research Center (IBBRC)

Supplementary Information

Supplementary Table 1. | One-Sample Test

			Test Value=0			
					95% Confidence interval of the difference	
**SH condition**	**t**	**df**	**Sig. (2-tailed)**	**Mean Difference**	**Lower**	**Upper**
1st Segment	-0.175	123	0.862	-0.0161	-0.1990	0.1668
2nd Segment	0.466	127	0.642	0.0406	-0.1319	0.2132
3rd Segment	-0.456	127	0.649	-0.0396	-0.2112	0.1321
4th Segment	-0.170	127	0.865	-0.0133	-0.1680	0.1415
5th Segment	0.214	127	0.831	0.0167	-0.1375	0.1708
6th Segment	-0.442	127	0.659	-0.0372	-0.2040	0.1296
**IH condition**						
1st Segment	-0.223	131	0.824	-0.0161616	-0.1598	0.1275
2nd Segment	0.028	131	0.977	0.0022727	-0.1560	0.1605
3rd Segment	-0.350	131	0.727	-0.0257576	-0.1714	0.1199
4th Segment	0.092	131	0.927	0.0070707	-0.1446	0.1587
5th Segment	-0.278	127	0.782	-0.0205729	-0.1672	0.1261
6th Segment	-0.293	131	0.770	-0.0214646	-0.1663	0.1233
